# Supplementary material for: Complexome profiling of the Chlamydomonas psb28 mutant reveals TEF5 as an early PSII assembly factor
Source: Plant Cell. 2025 Mar 17;37(6):koaf055. doi: 10.1093/plcell/koaf055 (PMC12164590; doi:10.1093/plcell/koaf055)
Supplement: koaf055_Supplementary_Data [file koaf055_supplementary_data.zip › psb28_interactive.zip!/plots/intensity/qualityPlots/peptides/CYP97C3_Cre08.g373100.t1.1.html]

### Cre08.g373100.t1.1

Trivial name: CYP97C3  
SeqCoverage [median]: 14.30  
Mass: 63.47 kDa  
  
 

| Sequence | MissedCleavage | Mass [Da] | wtIntensity | muIntensity | Score |
| --- | --- | --- | --- | --- | --- |
| AASAAAAAGTEYLNEADPSVLR | 0 | 2147.05 | 694.89 | 0.00 | 53.03 |
| ALEAVELIR | 0 | 1012.59 | 519.72 | 1991.73 | 83.27 |
| FGPLDGPVPNEQNTDFR | 0 | 1901.89 | 0.00 | 1445.32 | 66.50 |
| GAAASGSSGVAGGK | 0 | 1075.53 | 345.59 | 2037.23 | 143.50 |
| LSPTMADYGQLR | 0 | 1350.66 | 331.55 | 0.00 | 68.89 |
| LYPHPPVLLR | 0 | 1203.71 | 538.53 | 1387.61 | 57.96 |
|  |  |  | 2430.3 | 6861.9 |  |
